# Quantifying Hypertension and Cardiovascular Conditions in South Africa

**DOI:** 10.1007/s10935-025-00833-2

**Published:** 2025-03-24

**Authors:** Handan Wand, Sarita Naidoo, Vaneshree Govender, Jayajothi Moodley

**Affiliations:** 1 Biostatistics and Databases Program, Kirby Institute, University of New South Wales, Level 6, Wallace Wurth Building, Kensington, NSW 2052 Australia; 2 Discipline of Public Health Medicine, School of Nursing and Public Health, University of KwaZulu-Natal, Durban, South Africa; 3 The Aurum Institute, Johannesburg, South Africa

**Keywords:** Cardiovascular conditions, Hypertension, Obesity, South Africa, Population-level impact

## Abstract

South Africa has some of the highest prevalence and incidence rates of non-communicable diseases in the world. In this study, the burden of obesity and its impact on cardiovascular conditions such as hypertension were investigated among South African men and women. The study utilized data from the South African National Income Dynamics Study (SA-NIDS) surveys conducted from 2008 to 2017. A total of 80,270 individuals consented to participate, comprising 32,686 men (41%) and 47,584 women (59%). Besides multivariable logistic regression models, the relative importance of obesity on hypertension was assessed and compared to behavioral and socioeconomic conditions. Obesity and elevated waist-circumference measurements were the most prominent correlates of increased prevalence of hypertension. After adjusting for non-modifiable and background risk factors, the population-attributable risk (PAR%) of obesity on hypertension exceeded that of other traditional risk factors. It was substantially higher in women ($PAR\%s$ ranged: 52%, 95% CI: 50%, 55%) compared to men ($PAR\%s$ ranged: 33%, 95% CI: 31%, 36%). In sex-specific analyses, men and women with obesity were more than four and three times more likely, respectively, to be hypertensive (adjusted Odds Ratio (aOR): 4.41 and 3.72, *p* < 0.001) compared to those with normal weights. Prevention strategies targeting key modifiable factors such as obesity, waist circumference, smoking, alcohol use and lack of exercise, are likely the most effective means of identifying and reaching those at highest risk. Furthermore, developing and implementing socially and culturally appropriate awareness programs remain a research priority.

## Introduction

Hypertension is a major cause of cardiovascular-related diseases and estimated to be associated with 13% of the deaths globally (World Health Organisation (WHO), 2015; Clark et al., 2019). Number of hypertensive individuals continues to increase globally, with substantial country-level disparities (Akpa et al., 2020). Hypertension is estimated to be highest in African countries (WHO, 2016). Although, these rates vary across the continent, the proportion of hypertensive individuals has been reported to be as high as 69% in South Africa (Akpa et al., 2020; Lim et al., 2012). The country also has high obesity rates which is estimated to be as one of the highest in the world with significant gender-specific disparities (Aronow, 2017; Cois & Day, 2015). Obesity prevalence increased significantly from 23.5% in 2008 to 27.2% in 2012, with a higher prevalence among females (37.9%) compared to males (13.3%) in 2012 (*p*-value < 0.001). The overall BMI increase was + 1.57 kg/m^2^ per decade (95% CI: 0.93–2.22), higher in women (+ 1.82 kg/m^2^ per decade, 95% CI: 1.06–2.58) than in men (+ 1.03 kg/m^2^ per decade, 95% CI: 0.14–1.93) (Cois & Day, 2015). Furthermore, low socioeconomic conditions, and social and cultural beliefs have also been reported to be associated with hypertension (Alaba & Chola, 2013; Antignac et al., 2018; Sartorius et al., 2015).

As the burdens of comorbidities associated with non-communicable diseases continue their impact in South Africa, it is important to investigate their most influential contributors. The current study investigated the patterns of obesity, life-style factors and their interplay with high-blood pressure and other cardiometabolic conditions. In our study, we also estimated the proportion of hypertension associated with modifiable risk factors while adjusting for potential confounders. In a multivariable setting, some risk factors may be correlated with each other. Therefore, we employed a novel epidemiological technique (Spiegelman et al., 2007) to quantify the extent to which risk factors such as body mass index (BMI) and lifestyle behaviors influence hypertension rates, specifically defined as stage 1 (Carey et al., 2018; Greenland & Peterson, 2017) and stage 2 (Joint National Committee V, 1993) hypertension. Although there is an extensive research to report the predictors of hypertension and cardiometabolic conditions in various populations, their population-level burden in South African men and women are unknown.

## Materials and Methods

Cross-sectional data were collected from the South African National Income Dynamics Study (SA-NIDS) which was conducted during between 2008 and 2017. Details of these data sources were described elsewhere (http://www.nids.uct.ac.za/nidsdata). Survey participants were selected using a systematic random sample in a multistage stratified cluster sampling design setting. We analysed data from Black South Africans who comprised more than 90% of the participants in the five rounds of the cross-sectional South African National Income Dynamics Study (NIDS) surveys conducted in 2008 (NIDS Wave 1, 2018a), 2010–2011 (NIDS Wave 2, 2018b), 2012 (NIDS Wave 3, 2018c), 2014–2015 (NIDS Wave 4, 2018d), and 2017 (NIDS Wave, 2018e). Briefly, the surveys were conducted by the South African Labour and Development Research Unit based at the School of Economics (University of Cape Town). Study populations were selected from households using a two-stage random cluster sampling design from 52 district councils where clusters were household units. All the surveys had a similar aim and collected data to provide crucial information for unemployment, poverty and inequality in South Africa. Since NIDS was initiated, empirical findings from these data sources provided policy-makers and presented evidence for the key changes in social issues as well as unemployment, inequality and chronic poverty to education, health and the distribution of wealth.

### Measurements

Demographic and socioeconomic characteristics, including age, sex, language spoken at home, education (no schooling, primary, secondary (lower/upper) school, tertiary/technical education), religion, province of residence, employment status, medical aid/insurance, household income, characteristics of the residential areas, household conditions, and neighbourhood characteristics, were analysed. Additionally, lifestyle characteristics including smoking, alcohol use, and frequency of exercise were examined. Waist circumferences were categorized using traditional cut-off points. Specifically, we used the reference values recommended by the World Health Organization (WHO Consultation, 2008), which are also recommended for Black South African individuals. These values are (1) > 94 cm for men (45%) and > 80 cm for women (70%), and (2) > 102 cm for men (25%) and > 88 cm for women (54%). We used option 2 in our risk factor analysis. Body mass index (BMI) (kg/m^2^) was calculated using the participants’ height (cm) and weight (m^2^) measurements and categorised as follows: underweight (< 18.5 kg/m^2^), normal weight (18.5–24.9 kg/m^2^), overweight (25–29.9 kg/m^2^) and obese (30 + kg/m^2^) (WHO consultation, 2000).

### Primary Outcome Measurement

Trained fieldworkers measured survey participants' blood pressure at their residences using the “Omron M7 (HEM-780-E)”, an automated oscillometer upper arm blood pressure monitor suitable for both professional and home use. Following a 5-min rest period, both systolic and diastolic blood pressures were measured twice during each survey interview. Quality controllers ensured the completeness and accuracy of the data. The current study calculated the average of the two measurements (i.e., (first reading + second reading)/2) of systolic and diastolic pressures, which may potentially reduce variability and improve accuracy by minimizing the bias due to measurement errors.

In our analyses, we used two definitions for hypertension and conducted our primary analysis using the traditional definition of hypertension (i.e. Stage 2 hypertensive):

1. Stage 1 hypertensive: The 2017 American College of Cardiology /American Heart Association (2017 ACC/AHA) Guideline which classified individuals as hypertensive if the average values of the two measurements of systolic blood pressure ≥ 130 mmHg and/or diastolic blood pressure ≥ 80 mmHg (2017 ACC/AHA) (Carey et al., 2018; Greenland & Peterson, 2017).
2. Stage 2 hypertensive: the traditional definition which classified individuals as stage 1 hypertensive if the average values of the two measurements of systolic blood pressure ≥ 140 mmHg and/or diastolic blood pressure ≥ 90 mmHg (Joint National Committee V, 1993).

### Statistical Analysis

In our sex-specific analysis, the survey populations were described and formally compared by using the chi-square test. We first used multivariable logistic regression models which accounted for the multistage survey sampling nature of the design. The adjusted odds ratios were from multivariable models after adjusting the for the risk factors identified as significant correlates of hypertension status. The second part of the analysis focussed on population-level impacts of increased BMI and waist circumference on odds of hypertension as well as the other clinical endpoints using a multivariable version of the epidemiologic measure called population-attributable risk. In this approach, both characteristics, BMI and waist circumference categories were included in the model along with the other significant risk factors. In our analysis, we assessed potential multicollinearity between BMI and waist circumference. Using Variance Inflation Factor (VIF) calculations. A VIF value below 10 indicates that multicollinearity may not significantly distort the regression estimates. Models were assessed using the Hosmer–Lemeshow test-statistics.

### Estimating Population-Level Impacts of Risk Factors on Cardiovascular Conditions

An epidemiological measure called population attributable risk $(PAR\% )$ and its 95% CIs were calculated using a novel statistical approach after accounting for the associations between the predictors were also estimated (Spiegelman et al., 2007; Wand et al., 2019, 2023):

$$PAR\%$$$$= \frac{p(OR - 1)}{{p(OR - 1) + 1}} = 1 - \frac{1}{{\sum\nolimits_{s = 1}^{2} {p_{s} OR_{s} } }}$$$$s$$ indexes the levels of a risk factor of interest which was generalized to a multivariable model when there are several risk factors:

$$PAR\%$$$$_{{}} = \frac{{\sum\nolimits_{s = 1}^{S} {} p_{s} (OR_{s} - 1)}}{{1 + \sum\nolimits_{s = 1}^{S} {} p_{s} (OR_{s}^{{}} - 1) + 1}} = 1 - \frac{1}{{\sum\nolimits_{s = 1}^{S} {p_{s} OR_{s} } }}$$ where $$OR_{s}$$ and $$p_{s}$$, $$s = 1,...,S$$. The combinations of the prevalence’s of the several risk factors were considered, using the combination of exposures as well as ORs. Stata 16.0 (CS, TX, USA) and SAS 9.0 (SAS Inc., Cary, NC, USA) were used.

## Results

Overall, 32,686 (41%) men and 47,584 (59%) women enrolled in NIDS surveys during the period of 2008–2017 (Table 1). The study participants were described and compared by sex. Women were significantly older than men (median age: 35 vs. 29 years old) and more likely to be single/not cohabiting. A higher proportion of women had no education (11% vs. 7%) and no regular income (80% vs. 71%) compared to men. Approximately one third of the population was residing in the province of KwaZulu-Natal.

**Table 1:** Socio-economic Characteristics of the study population by gender:

Characteristics	Overall N = 80,270 (100%)	Males N = 32,686 (41%)	Females N = 47,584 (59%)	*p*-value^§^
*Age (median, IQR)*	32 (22–49)	29 (21–45)	35 (23–51)	< 0.001
< 20 years	16%	19%	14%	< 0.001
20–24 years	15%	17%	14%	
25–29 years	13%	14%	12%	
30–34 years	10%	10%	10%	
35–39 years	8%	8%	8%	
40–44 years	7%	6%	7%	
45–49 years	6%	7%	7%	
50–55 years	5%	6%	6%	
55–59 years	4%	6%	5%	
60 + years	10%	15%	13%	
*Marital status*				< 0.001
Single/not cohabiting	74%	71%	75%	
Married/cohabiting	26%	29%	25%	
*Education levels*				< 0.001
No education	10%	7%	11%	
Primary	2%	1%	2%	
Lower Secondary	8%	7%	8%	
Upper Secondary	30%	34%	28%	
Tertiary/Technical/Other	37%	36%	38%	
*Language spoken at home*				< 0.001
IsiXhosa	10%	17%	5%	
IsiZulu	78%	70%	84%	
Afrikaans	10%	11%	9%	
English	2%	2%	2%	
*Province of residence*				< 0.001
Other provinces	67%	66%	69%	
KwaZulu-Natal	33%	34%	31%	
Religion				< 0.001
None	10%	17%	5%	
Christian	78%	70%	84%	
African religion	10%	11%	9%	
Other	2%	2%	2%	
*Employed/regular salary*				< 0.001
No	76%	71%	80%	
Yes	24%	29%	20%	
*Covered by medical aid*				< 0.001
No	93%	92%	94%	
Yes	7%	8%	6%	
*Household income*				< 0.001
< 1500	28%	26%	28%	
1500- < 3000	30%	29%	32%	
3000- < 4500	17%	17%	17%	
4500 +	25%	28%	23%	
*Household conditions*				
Structured house	57%	56%	57%	0.145
Running water	33%	34%	32%	< 0.001
Electricity	82%	82%	81%	0.041
Toilet	40%	42%	38%	< 0.001
*Neighbourhood conditions*				
Frequent drug-related crime	72%	72%	72%	0.465
Frequent burglary/theft	38%	38%	38%	0.661
Frequent domestic violence	22%	22%	22%	0.269

A significantly higher proportion of men reported being regular cigarette smokers compared to women (29% vs. 3%); alcohol intake (3 + /week) was higher in men (Table 2). In women, 80% of them did never exercise, this proportion was 57% among men. Median BMI was 22.0 (IQR: 19.8, 25.5) kg/m^2^ for men and 27.3 (IQR: 23, 32.8) kg/m^2^ for women. Overall, 65% of women were overweight (or obese) compared to men (31%). Women also had significantly higher waist-circumference compared to men (70% vs. 45%, *p* < 0.001).

**Table 2:** Life-style and Health status indicators by gender:

Characteristics	Overall N = 80,270 (100%)	Males N = 32,686 (41%)	Females N = 47,584 (59%)	*p*-value^§^
*Survey rounds*				0.100
Wave 1 (2008)	15%	15%	15%	
Wave 2 (2010–2011)	18%	19%	18%	
Wave 3 (2012)	19%	19%	19%	
Wave 4 (2014–2015)	24%	24%	23%	
Wave 5 (2017)	24%	24%	24%	
*Smoke regularly*				< 0.001
No	87%	71%	97%	
Yes	13%	29%	3%	
*Alcohol intake*				< 0.001
Never/quit drinking	59%	47%	68%	
Rarely/some /week	14%	24%	7%	
3 + day/week	2%	4%	1%	
No response	25%	25%	25%	
*Frequency of exercise/week*				< 0.001
Never	72%	57%	83%	
Less than once	7%	8%	5%	
Once/twice	10%	15%	7%	
Three time or more	11%	20%	5%	
*Ever tested for HIV?*				< 0.001
No	42%	50%	34%	
Yes	58%	50%	66%	
	*Study measurements*			
BMI (median, IQR)	24.8 (21.1, 30.3)	22.0 (19.8–25.5)	27.3 (23.0–32.8)	< 0.001
Normal weight (18.5- < 25 kg/m^2^)	49%	69%	35%	
Overweight (25–29 kg/m^2^)	22%	18%	25%	
Obese (30 + kg/m^2^)	29%	13%	40%	
*Waist circumference (cm)*^*ξ*^				< 0.001
High waist circumference (men > 94, women > 80)	58%	45%	70%	
High waist circumference (men > 102, women > 88)	40%	25%	54%	
Pulse per minute (median, IQR)	75 (67–85)	71 (63–80)	78 (70–87)	< 0.001
Low (< 60)	10%	17%	5%	< 0.001
Normal (60–100)	87%	81%	90%	
High (> 100)	4%	3%	5%	
*Diastolic blood pressure*				
> 80 mmHg	46%	45%	47%	< 0.001
> 90 mmHg	30%	29%	31%	< 0.001
*Systolic blood pressure*				
> 130 mmHg	30%	33%	28%	< 0.001
> 140 mmHg	23%	24%	22%	< 0.001
Hypertension^¶¶^ (Stage 1)				0.020
No	49%	49%	50%	
Yes	51%	51%	50%	
*Hypertension*^*¶*^* (Stage 2*)				0.950
No	64%	64%	64%	
Yes	36%	36%	36%	

^¶^diastolic/systolic > 140/ > 90; ^¶¶^diastolic/systolic > 130/ > 80; ^§^*p*-values: for the categorical variables are from chi-square test and continuous variables are from rank-sum test; ^ξ^ Traditional cut-off values (WHO Consultation, 2008)

### Age-BMI Specific Hypertension Prevalence

**Fig. 1:**
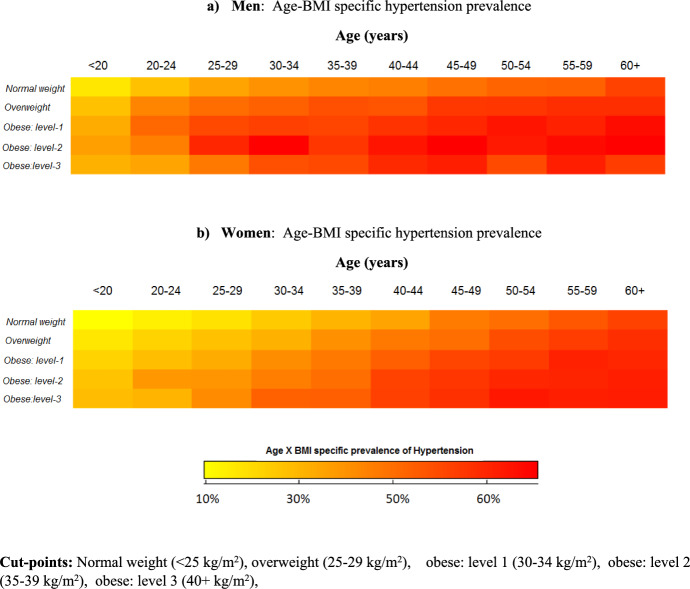
Age-BMI specific hypertension prevalence

In additional analyses, BMI x age (in deciles) specific hypertension prevalences were quantified using a three-dimensional “*heat-map*” by gender where Fig. 1a (men) and b (women). These data visualizations showed that higher proportion of hypertension in older age groups in both sexes. Comparison of these two figures revealed gender-specific differences and similarities in prevalence of hypertension by the BMI categories; while younger men had significantly higher odds to be hypertensive compared to women at the same age, these differences appeared to be diminished in older age groups.

### Correlates of Hypertension

In multivariable logistic regression models, the odds of developing hypertension were significantly higher in older age groups compared to individuals younger than 20 years of age, with aORs ranging from 1.76 (20–24 years) to 10.14 (60 + years old) for men and 1.50 (20–24 years old) to 15.54 (60 + years old) for women (P_trend_ < 0.001, both) (Table 3). Overweight/obese were significantly more likely to be hypertensive (aORs: 1.71 and 4.41 for men; aORs: 1.16 and 3.71 for women). Similar associations were observed with increasing levels of waist measurements and hypertension (aORs: 3.44 and 2.75 for men and women). No schooling was also correlated with higher odds of hypertension in both sexes, while unemployment was a significant correlate of hypertension in women only. Men and women who indicated that they never exercise was more than 50% more likely to be hypertensive. Participants who indicated using alcohol at least three days per week (aOR: 1.69 (men) and 1.74 for (women)), and regular cigarette smokers were also more likely to be hypertensive compared to those who did not smoke (aOR: 1.21 (men) and 1.67 (women) respectively).

**Table 3:** Sex-specific correlates of hypertension and PAR% (95% CI): Results from multivariable models

	Men			Women		
	Adjusted OR^Ɛ^ (95% CI)	p-value	PAR% (95% CI)	Adjusted OR^Ɛ^ (95% CI)	p-value	PAR% (95% CI)
*Age groups*						
< 20 years	1		Not modifiable	1		Not modifiable
20–24 years	1.76 (1.60, 1.94)	< 0.001		1.50 (1.33, 1.65)	< 0.001	
25–29 years	2.63 (2.34, 2.89)	< 0.001		2.01 (1.82, 2.22)	< 0.001	
30–34 years	3.27 (2.91, 3.60)	< 0.001		3.00 (2.70, 3.30)	< 0.001	
35–39 years	3.86 (3.45, 4.31)	< 0.001		4.14 (3.72, 4.60)	< 0.001	
40–45 years	4.50 (4.01, 5.05)	< 0.001		6.02 (5.43, 6.68)	< 0.001	
45–49 years	5.76 (5.12, 6.48)	< 0.001		7.88 (7.10, 8.73)	< 0.001	
50–54 years	7.00 (6.21, 7.91)	< 0.001		10.01 (8.99, 11.12)	< 0.001	
55–59 years	7.42 (6.53, 8.43)	< 0.001		12.84 (11.48, 14.34)	< 0.001	
60 + years	10.14 (9.18, 11.23)	< 0.001		15.54 (14.16, 17.04)	< 0.001	
*Education*						
Tertiary/Technical	1			1		
Secondary (lower/upper)	1.40 (1.27, 1.75)	< 0.001	19% (13%, 22%)	1.10 (1.06, 1.15)	< 0.001	26% (24%, 29%)
Primary	2.00 (1.63, 2.44)	< 0.001		2.74 (2.35, 3.19)	< 0.001	
No schooling	2.37 (2.15, 2.61)	< 0.001		3.55 (3.32, 3.80)	< 0.001	
Employed/income			*			16% (15%, 18%)
No	0.95 (0.88, 1.01)	0.860		1.31 (1.22, 1.37)	< 0.001	
Yes	1			1		
*Marital status*						
Single/not cohabitating	1			1		
Married/cohabitating	2.39 (2.28, 2.45)	< 0.001	29% (26%, 31%)	1.76 (1.68, 1.83)	< 0.001	16% (14%, 18%)
*Smoke cigarettes*						
No	1			1		
Yes	1.21 (1.14, 1.27)	< 0.001	6% (5%, 7%)	1.67 (1.49, 1.87)	< 0.001	3% (1%, 5%)
*Alcohol intake*						
Never	1		9% (7%, 11%)	1		1% (0%, 3%)
Rarely/some	1.23 (1.16, 1.33)	< 0.001		1.04 (0.98, 1.13)	0.651	
3 + days/week	1.69 (1.48, 1.83)	< 0.001		1.76 (1.32, 2.18)	< 0.001	
*BMI categories*^*0*^						
Normal weight	1			1		
Overweight	1.71 (1.57, 1.87)	< 0.001	33% (31%, 36%)	1.16 (1.02, 1.40)	< 0.001	52% (50%, 55%)
Obese	4.41 (4.02, 4.84)	< 0.001		3.72 (3.42, 3.06)	< 0.001	
*Waist-circumference*						
Normal^1^	1			1		
High^2^	3.44 (3.24, 3.66)	< 0.001	29% (26%, 32%)	2.75 (2.63, 2.88)	< 0.001	55% (53%, 57%)
*Exercise/week*						
At least once a week	1			1		
Never	1.60 (1.54, 1.70)	< 0.001	23% (21%, 27%)	1.64 (1.55, 1.75)	< 0.001	33% (30%, 36%)

^*^not applicable; ^Ɛ^ the models were adjusted for survey rounds; ^0^Normal weight: < 25 kg/m^2^; Overweight: 25–29 kg/m^2^; Obese: 30 + kg/m^2^;^1^ < 80 cm for women and < 94 cm for men; ^2^ > 80–88 cm for women and > 94–102 cm for men;

### Population-Level Estimates for Cardiovascular Conditions

Higher BMI and waist circumference were both determined as the substantial contributors of increased prevalence of hypertension regardless of sex (Table 4). The PAR% of overweight/obesity on hypertension was estimated as 36% (95% CI: 34%, 40%) in men; this proportion was 60% (95% CI: 57%, 63%) in women. We interpreted the non-overlapping confidence intervals as statistically significant difference by sex. Consistent with these results, increasing waist circumference also had a substantial impact on being hypertensive with PAR%: 29% and 51% for men and women, respectively. These two anthropometric measures collectively accounted for 36% and 59% of the hypertension cases in men and women respectively; while combined impact of smoking and exercise was broadly similar with 27% and 34% for men and women, respectively. Smoking and alcohol intake were minimal with PAR%: 6% and 10% for men; 2% and 1% for women.

**Table 4:** Gender-specific population-attributable risks and 95% CI of modifiable risk factors on hypertension prevention:

	Combined impact of modifiable risk factors: PAR% (95% CI)	
	Men	Women
Full PAR% ^ǂ^	59% (56%, 62%)	76% (71%, 81%)
*Anthropometric measures*		
Overweight + Obese + Waist circumference	36% (34%, 40%)	59% (50%, 61%)
*Behavioural factors*		
Smoking + alcohol intake + exercise	27% (24%, 29%)	34% (31%, 36%)
*Socioeconomic factors*		
Lack of education + unemployment^§^	17% (11%, 20%)	29% (25%, 33%)
*Anthropometric measures + Life style factors *		
Overweight + Obese + Waist circumference + Smoking + alcohol + exercise	54% (51%, 58%)	68% (65%, 71%)

^§^ aOR < 1, therefore did not contribute for males; ^ǂ^ when all the factors were considered; results were further adjusted for age, survey year and marital status

**Fig. 2:**
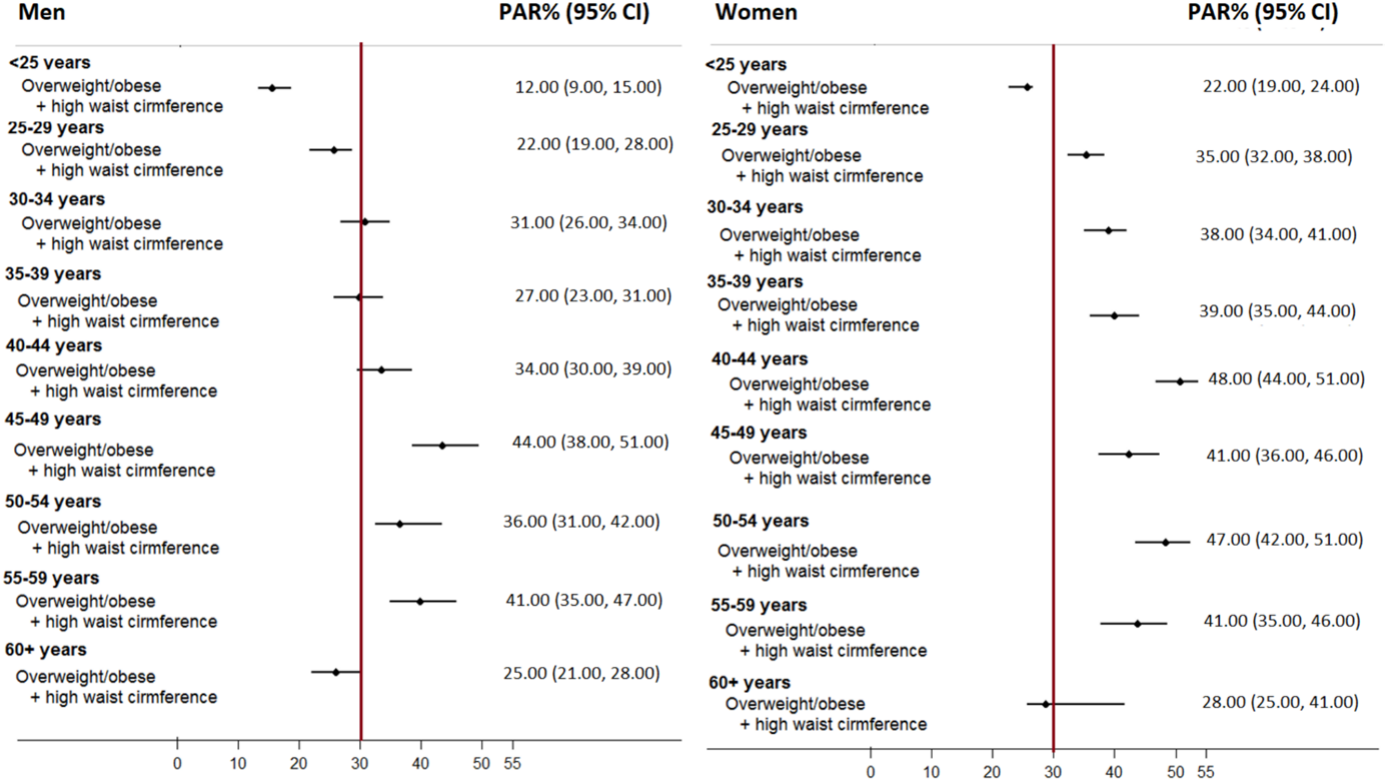
Age-stratified gender specific combined impacts of overweight/obese and waist circumference for hypertension

In an age-stratified analysis (Fig. 2), combined impact of higher BMI and waist circumference measures collectively accounted for 12% to 41% of the hypertension among men, with the highest impact observed in the 45–49-year age group of. While these two factors had significantly higher impact in women, associated PAR%s ranged from 22 to 48%, with the largest impact in the 40–44-year age group. Our tests for multicollinearity, where VIF were calculated as 8.52 for men and 8.84 for women, indicating that while there is a strong monotonic relationship between BMI and waist circumference (correlation coefficients of 68% for men and 80% for women), multicollinearity did not significantly affect the outcomes of our regression analysis. This confirms that both BMI and waist circumference can be retained as significant predictors in our multivariable model without causing instability due to multicollinearity.

## Discussion

In our study, obesity and elevated waist-circumference measurements were identified as the most influential correlates of increased prevalence of hypertension and risk of developing cardiovascular diseases. After adjusting for non-modifiable factors such as age and the background risk factors, such as marital status, individual-level impacts of body composition measurements exceeded that of other risk factors which was substantially higher in women. This was due to the higher prevalence of obesity and elevated waist-circumference in women (65 and 70% respectively) than in men (31 and 45% respectively). Their combined impacts were 68% in women and 54% in men. Sex-specific disparities sustained in age-stratified analyses where 25–51% of the hypertension cases were collectively attributed to the increased levels of BMI and waist circumference in women, compared to 15% to 44% in men. As reported previously, excess body fat (overweight or obesity) is an established risk factor for hypertension (Akpa et al., 2020). We observed high rates of obesity in this population where 40% of the women were categorised as obese which is one of the highest female obesity rates reported in the region. Although there is an extensive research to report strong associations between excess body fat and hypertension in other populations, their population-level impacts are unknown in South African (Akpa et al., 2020; Case & Menendez, 2009; Micklesfield et al., 2013).

Further, our results also highlighted gender-specific disparities in younger age groups. In the nationally representative surveys, 38% of the South African women were older than 50 years of age, were estimated to be obese (Cois & Day, 2015; Sartorius et al., 2015). This figure agrees with our estimate who were much younger with average age of 35 (Akpa et al., 2020). In our study, proportion of hypertension was significantly higher among < 30 years of age compared to women in the same age group (40% vs. 29% men and women, respectively). These disparities appeared to be diminished in older age groups with hypertension rates exceeding that of 80%, and observed in aged > 50 years of age in both sex. Our findings aligned with previous research. Hypertension was frequently reported to be more common in younger men than younger women which was primarily attributed to the male-gender androgen related abnormalities (Kayima et al., 2015; Quinkler et al., 2005).

Consistent with previous research, reporting no exercise is also more common in hypertensive group with moderate impact of $PAR\%:$ 23% (men) and 33% (women), respectively (Gebreselassie et al., 2015; Kruger et al., 2012). Although more prevalent in men compared to women, smoking was not a big contributor to hypertension ($PAR\%$: 6% and 2% for men and women, respectively). Despite significant odds ratios associated with frequent alcohol intake, only 10% (men) and 1% (women) of the cases were attributed to this characteristic which were also attributed to very low proportion of men and women who reported to be frequent drinkers (< 5%).

Low socioeconomic indicators, including lack of schooling and unemployment had a moderate impact on hypertension with $PAR\%:$ 30% in women compared to $PAR\%:$ 10% in men. This gender-specific disparity was primarily due to the high unemployment and low-levels of education in women compared to men. As reported in nationally representative studies, South African women continue to be the most vulnerable group for poverty compared to men (Wand et al., 2019). According to the South African census data, fifty percent of the households are headed by single females who were also more likely to be exposed to low-socioeconomic conditions for various reasons (Echouffo-Tcheugui et al., 2015; Puoane et al., 2002). A significant negative association was previously reported between increasing individual-wealth index and risk of hypertension in other populations worldwide (Dzudie et al., 2017). In addition to this, compared to the high-income countries, prevalence of hypertension was reported to be significantly higher in low-income countries (Echouffo-Tcheugui et al., 2015).

After many years of efforts to reduce the burden of hypertension in the region, the current study reported one of the highest hypertension rates in this nationally representative sample of South Africans (Dzudie et al., 2017). Overall, 50% of the survey population had high blood pressure (stage 1); while this proportion exceeded 60% among those aged 25 years or older (data not shown) compared to 46% in 20 African countries who participated in WHO STEPS (STEPwise approach to surveillance) surveys conducted. These estimates are alarming and have significant adverse health implications.

High obesity rates in South Africa may have implications exclusive to this population, largely influenced by unique social and cultural beliefs. As previously reported, a higher body mass is often seen as an indicator of good health, prosperity, and social status, particularly among black South African women (Puoane et al., 2005; Matoti-Mvalo & Puoane, 2011; Pedro et al., 2016). These references are illustrative of the cultural beliefs and are used to contextualize attitudes towards body mass within the South African setting. They are intended to demonstrate associative trends rather than establish causality. Additionally, South Africa has the highest number of HIV-infected individuals globally, and thinness is frequently associated with HIV/AIDS. Consequently, many women prefer to maintain a higher body weight to avoid stigma related to the disease. Understanding these cultural aspects is crucial for designing effective health interventions that are culturally sensitive and can lead to substantial health improvements within South African communities.

As the burden of obesity and cardiovascular conditions increases in the region, South Africa also has the highest number of HIV infections globally (UNAIDS, 2021, 2022). Besides increasing awareness of safe sexual behaviours, urgent strategies are needed to prevent obesity and obesity related adverse cardiovascular conditions including hypertension among South Africans. In 2004, African Union member states declared hypertension as being the greatest health challenge faced African countries (Dzudie et al., 2017), after HIV/AIDS, and called for affordable and effective screening and treatment programs across the continent. Preventing non-communicable diseases were included in WHO’s 2013 to 2020 global action plan which aimed to reduce hypertension by 25% in 2020 (World Health Organization, 2013). In 2014, practical guidelines developed by a group of expert who were the members of The Pan-African Society of Cardiology (PASCAR) (Dzudie et al., 2017). Their action plans included how to develop effective detection, treatment and control strategies in order to reduce hypertensive individuals by 25% by 2025. Despite all these efforts to reduce hypertension and obesity in African countries, the global data suggest significant increases in the region (Akpa et al., 2020).

## Limitations

The current study have the following limitations: (1) This study is cross-sectional, inheriting the typical limitations associated with this design. It can only show associations rather than causation, limiting our ability to infer directional relationships between variables. (2) While anthropometric and characteristics were collected by study personnel, behavioral and sociodemographic characteristics were self-reported by the survey participants, introducing potential reporting bias. (3) We focused exclusively on data from Black South Africans, as they represented the majority (94%) of the survey population. This focus enhances the robustness and national representativeness of our findings within this demographic, though it limits generalizability across all South African ethnic groups. (4) Additionally, since national surveys are conducted periodically, there may be temporal gaps that fail to capture changes in behaviors or emerging trends between survey cycles. (5) Finally, we used the previously established BMI cut-off points for classifying obesity (BMI: 30 + kg/m^2^), which were derived for different populations and may have different implications for Black South Africans. Despite this, these cut-off points demonstrated better discriminative power compared to those suggested for Black South Africans in other studies (data not shown).

Despite these limitations, combined survey population provided a unique opportunity to investigate the population level-impact of socioeconomic conditions, behavioral factors as well as excess body on hypertension.

## Conclusion

Given the significant impact of treatment programs in cardiovascular disease prevention and control, it is important to identify and target the most vulnerable populations who are at highest risk of hypertension. Our findings demonstrated that high proportion of hypertension cases could potentially be prevented by targeting individuals with excess body fat even if the other established risk factors remained unchanged. While the effects of diet on hypertension were not directly assessed in this study, we provided evidence of a lack of physical activity, which can serve as a surrogate for several established risk factors including obesity and hypertension, both of which contribute to cardiometabolic diseases. Integrating healthy diet and lifestyle messages into existing HIV/AIDS prevention programs could be both an effective and cost-efficient approach to improving overall health and well-being. Furthermore, the development of community-level programs in various settings, such as schools and workplaces, could promote balanced nutrition, regular physical activity, and smoking cessation. These programs would emphasize reducing processed foods and increasing the consumption of fresh fruits and vegetables. By encouraging healthier behaviours at both individual and community levels, such initiatives could play a crucial role in preventing obesity, hypertension, and related cardiovascular diseases.

## Data Availability

Data are publicly available from the South African National Income Dynamics Study (SA-NIDS) https://www.datafirst.uct.ac.za/.
